# Complete mitochondrial genome of fire-tailed myzornis (*Myzornis pyrrhoura*) and white-browed fulvetta (*Fulvetta vinipectus*)

**DOI:** 10.1080/23802359.2020.1814884

**Published:** 2020-09-08

**Authors:** Danjie Li, Shimiao Shao, Xiaoyi Wang, Xinran Wu, Xu Luo

**Affiliations:** Key Laboratory for Conserving Wildlife with Small Populations in Yunnan/Faculty of Biodiversity and Conservation, Southwest Forestry University, Kunming, China

**Keywords:** *Myzornis pyrrhoura*, *Fulvetta vinipectus*, mitogenome, phylogeny

## Abstract

In this study, the complete mitochondrial genomes of *Myzornis pyrrhoura* and *Fulvetta vinipectus* were sequenced and described for the first time. The whole mitochondrial genomes of *M. pyrrhoura* and *F. vinipectus* are 17,397 bp and 16,961 bp in length, with the G + C percentage 46.34% and 47.36%, respectively. Both genomes contain 13 protein-coding genes, 22 transfer RNA genes, 2 ribosome RNA genes, and 1 non-coding control region. The arrangement of genes is identical to mitochondrial genomes of Sylviidae species reported previously. A phylogenetic reconstruction supported that *M. pyrrhoura* and *F. vinipectus* are members of family Sylviidae. The mitochondrial genomes of these two species reported here would be helpful in better understanding the phylogeny and evolution of Sylviidae.

## Introduction

The fire-tailed Myzornis (*Myzornis pyrrhoura*) and white-browed fulvetta (*Fulvetta vinipectus*) (Aves, Passeriformes, Sylviidae) are two species of small-sized sylviid babblers, which are both rated Least Concern on the IUCN Red List (IUCN 2018). *Myzornis pyrrhoura* has bright green color with black mask and crown scalloping, having red flash in black-and-white wing, red-sided tail, and rather long, thin, slightly decurved black bill (Josep et al. 2007). *Fulvetta vinipectus* is a small fulvetta in soft rich browns and ochrous-buffs with pale eye in dark mask and bold white supercilium (Josep et al. 2007). They are both resident birds and sympatric breeders above 2700 m in Gaoligong Mountains (Wang et al. 2016; Liang et al. 2017). The samples were collected around Pianma Pass (25°58′–26°03′N, 98°41′–98°44′E; 3000–3800 m), which is located in the middle section of Gaoligong Mountains (Liang et al. 2017). The two specimens *M. pyrrhoura* and *F. vinipectus* are deposited in the Zoological Collection Center of Southwest Forestry University, under the voucher number of SWFU-b0466 and SWFU-b0470.

## Methods

The total mitochondrial DNA was extracted from the muscle tissue and was sequenced using next-generation sequencing. The entire mitochondrial genome sequences of *M. pyrrhoura* and *F. vinipectus* have been deposited under the GenBank accession numbers MK612108 and MT263986.

**Figure 1.**
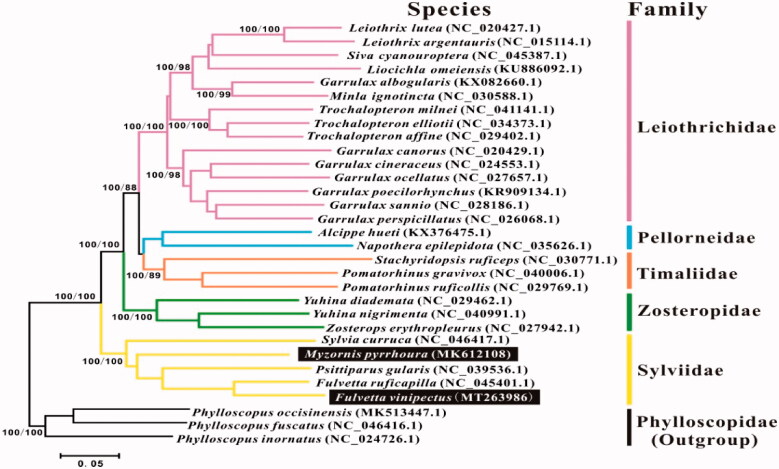
The phylogenetic tree based on combined protein-coding gene sequences of *F. vinipectus* and *M. pyrrhoura* and 26 relative species. The numbers following the species names are GenBank accession numbers. Numbers at a node refer to maximum parsimony (BP, left) and Bayesian posterior probability (BPP, right).

Phylogenetic tree of these two species among babblers were presented by maximum parsimony (MP) analyses using PAUP 4.0b10 software with 1000 bootstrap replication (Swofford 2002). Bayesian inference was calculated with MrBayes3.1.2. A general time-reversible (GTR) model of DNA substitution (Rodríguez et al. 1990) and a proportion of invariant sites and unequal rates among sites were modeled with a gamma distribution (GTR + I+G) (Yang 1996; Ronquist and Huelsenbeck 2003). Four Markov chains were run for 2,000,000 generations until the average standard deviation (SD) was less than 0.01. Sequences of other babblers including *Pomatorhinus gravivox*, *Stachyridopsis ruficeps*, and *Pomatorhinus ruficollis*, etc., were obtained from GenBank (Figure 1). Three Phylloscopidae species were used as the outgroup following Cai et al. (2019) and Gelang et al. (2009).

## Results

The complete mitochondrial genomes of *M. pyrrhoura* and *F. vinipectus* are 17,397 bp and 16,961 bp in length, respectively. The base composition of *M. pyrrhoura* mitochondrial genome G + C percentage was 46.34% and *F. vinipectus* 47.36%. Each of them consists of 13 protein coding, two rRNA, 22 tRNA genes, and two non-coding control regions (D-loop). The components and arrangement of genes were the same as those of other babblers species reported previously, such as *Garrulax affinis* (Huang et al. 2016) and *Phylloscopus inornatus* (Qing et al. 2015). For both *M. pyrrhoura* and *F. vinipectus*, COI and ND3 are initiated with ‘ATT’ and ‘ATA’, respectively. While the remaining 11 protein-coding genes begin with an ‘ATG’ start codon. Protein-coding genes of *M. pyrrhoura* were stopped by four types of complete stop codons (TAA for ND1, ND2, ND3, ND4L, COII, ATP6 and ATP8, AGA for ND5, AGG for COI, TAG for Cytb and ND6) and one incomplete stop codon (T for COIII and ND4), while protein-coding genes of *F. vinipectus* were stopped by the same five types of complete stop codons but with different arrangement (TAA for ND2, ND3, ND4L, ND5, Cytb, COII, ATP6 and ATP8, AGA for ND1, AGG for COI, TAG for ND6) and one incomplete stop codon (T for COIII and ND4). ND6 and eight tRNA genes, i.e. tRNA^Gln^, tRNA^Ala^, tRNA^Asn^, tRNA^Cys^, tRNA^Tyr^, tRNA^Ser^, tRNA^Pro^, and tRNA^Glu^, were encoded on a light strand (L-strand), while all of the remaining genes were located on the heavy strand (H-strand). The two control regions of mitochondrial genome were D-loop1 (1154 bp in *M. pyrrhoura* and 1144 bp in *F. vinipectus*) and D-loop2 (657 bp in *M. pyrrhoura* and 245 bp in *F. vinipectus*).

The reconstructed phylogenetic tree supported the placement of *M. pyrrhoura* and *F. vinipectus* in the Sylviidae family (Figure 1). All of the nodes were strongly supported by the MP and Bayesian analysis. This study identified *F. vinipectus* as a close relative of *F. ruficapilla*, which was also supported by BI analysis results of Cai et al. (2019). Nevertheless, *M. pyrrhoura* is an unpaired branch separating from the other remaining species in the Sylviidae family, that was also consistent with the result of Cai et al. (2019) and Gelang et al. (2009). The mitochondrial genomes reported here would be useful in the current understanding of the phylogeny and evolution of Sylviidae.

## Data Availability

The entire mitochondrial genome sequences of *M. pyrrhoura* and *F. vinipectus* have been deposited under the GenBank accession numbers MK612108 and MT263986, which are available at https://www.ncbi.nlm.nih.gov.
